# Influence of Short-Term Glucocorticoid Therapy on Regulatory T Cells *In Vivo*

**DOI:** 10.1371/journal.pone.0024345

**Published:** 2011-09-02

**Authors:** Silviu Sbiera, Thomas Dexneit, Sybille D. Reichardt, Kai D. Michel, Jens van den Brandt, Sebastian Schmull, Luitgard Kraus, Melanie Beyer, Robert Mlynski, Sebastian Wortmann, Bruno Allolio, Holger M. Reichardt, Martin Fassnacht

**Affiliations:** 1 Endocrinology and Diabetology Unit, Department of Internal Medicine I, University Hospital, University of Würzburg, Würzburg, Germany; 2 Department of Cellular and Molecular Immunology, University of Göttingen Medical School, Göttingen, Germany; 3 Department of Oto-Rhino-Laryngology, University of Würzburg, Würzburg, Germany; University of Muenster, Germany

## Abstract

**Background:**

Pre- and early clinical studies on patients with autoimmune diseases suggested that induction of regulatory T(T_reg_) cells may contribute to the immunosuppressive effects of glucocorticoids(GCs).

**Objective:**

We readdressed the influence of GC therapy on T_reg_ cells in immunocompetent human subjects and naïve mice.

**Methods:**

Mice were treated with increasing doses of intravenous dexamethasone followed by oral taper, and T_reg_ cells in spleen and blood were analyzed by FACS. Sixteen patients with sudden hearing loss but without an inflammatory disease received high-dose intravenous prednisolone followed by stepwise dose reduction to low oral prednisolone. Peripheral blood T_reg_ cells were analyzed prior and after a 14 day GC therapy based on different markers.

**Results:**

Repeated GC administration to mice for three days dose-dependently decreased the absolute numbers of T_reg_ cells in blood (100 mg dexamethasone/kg body weight: 2.8±1.8×10^4^ cells/ml vs. 33±11×10^4^ in control mice) and spleen (dexamethasone: 2.8±1.9×10^5^/spleen vs. 95±22×10^5^/spleen in control mice), which slowly recovered after 14 days taper in spleen but not in blood. The relative frequency of FOXP3^+^ T_reg_ cells amongst the CD4^+^ T cells also decreased in a dose dependent manner with the effect being more pronounced in blood than in spleen. The suppressive capacity of T_reg_ cells was unaltered by GC treatment *in vitro*. In immunocompetent humans, GCs induced mild T cell lymphocytosis. However, it did not change the relative frequency of circulating T_reg_ cells in a relevant manner, although there was some variation depending on the definition of the T_reg_ cells (FOXP3^+^: 4.0±1.5% vs 3.4±1.5%*; AITR^+^: 0.6±0.4 vs 0.5±0.3%, CD127^low^: 4.0±1.3 vs 5.0±3.0%* and CTLA4+: 13.8±11.5 vs 15.6±12.5%; * p<0.05).

**Conclusion:**

Short-term GC therapy does not induce the hitherto supposed increase in circulating T_reg_ cell frequency, neither in immunocompetent humans nor in mice. Thus, it is questionable that the clinical efficacy of GCs is achieved by modulating T_reg_ cell numbers.

## Introduction

Glucocorticoids (GCs) are a class of steroid hormones that bind to the glucocorticoid receptor, which is present in almost every vertebrate animal cell [1]. This explains the large variety of physiological roles played by GCs [2], [3]. They are crucial modifier of the metabolic [4] and immune system [5], [6], but are also important for development [7] as well as for arousal and cognition [8]. During an immune response, GCs contribute to the termination of inflammation by both suppressing and enhancing activities of the immune system [9]. At the level of the innate immune system GCs induce neutrophilia by increasing polymorphonuclear cell release from the bone marrow while inhibiting their transmigration to inflammatory sites [10], [11]. At the same time they induce apoptosis of basophils and eosinophils [12], [13]. In a similar way GCs control the movement of circulating monocytes while enhancing the phagocytotic ability and antigen uptake by the tissue macrophages and therefore speed up the clearance of foreign antigens and microorganisms [14]. At the level of leukocyte gene expression, a huge number of proinflammatory cytokines (IL1β, TNFα, IL-6, IL-8, IL-12, IL-18 etc) and chemokines (both CC and CXC) is strongly suppressed by GCs, while the anti-inflammatory cytokines IL-10 and TGFβ are upregulated [15]. On the other hand, GCs favor antibody production by promoting the generation of immunoglobulin secreting plasma cells [16]. Accordingly, GCs promote the polarization of naïve T-cells to a Th2 phenotype while at the same time promoting an immature dendritic cell phenotype [17]. Both effects may be in part responsible for a presumed upregulation of regulatory T (T_reg_) cells by GCs [18]. In the last decades, it became clear that T_reg_ cells play an essential role in self-tolerance and for maintaining immune system homeostasis [19].

Failure of self-tolerance often leads to autoimmune diseases (AD), with an incidence of 5–10 % of the population [20]. Emergence of autoimmune diseases is not completely understood, it is however documented that T cells often play an essential role in their development (e.g. diabetes mellitus type I or type A gastritis [21]). Some of these autoimmune diseases are probably triggered by infections (e.g. Guillian Barré syndrome [22], [23]), whereas in others it has been shown that tolerance defects are due to a decrease in number or defective expression pattern in the T_reg_ cell population [24], [25]. Since more than 60 years GCs are often the first line therapy in inflammatory and autoimmune responses [26] and around 30 years ago a connection between GCs and a “suppressor” T cell phenotype was initially shown *in vitro* [27], [28]. In the meantime, a number of different T_reg_ cell populations have been described [25], [29], of which the most studied one expresses CD4 and high levels of the IL-2 receptor alpha-chain (CD25) [19], [30]. Several additional surface markers have been reported to discriminate naturally occurring T_reg_ cells from other activated CD4^+^ Th2 cells such as cytotoxic T-lymphocyte antigen 4 (CTLA4) [31], [32], the nuclear transcription factor forkhead box P3 (FOXP3), the activation-inducible tumor necrosis factor receptor AITR [33] and low expression of the IL-7 receptor (CD127) [34], [35]. Although expression of FOXP3 is considered as the best tool to define T_reg_ cells by many authors [36], [37], [38], even this marker (as all other markers) is not specific to human T_reg_ cells and can be transiently induced on all effector T cell populations upon activation.

In 2004, Karagiannidis *et al.* showed for the first time that GC treatment *in vivo* (both systemic and inhaled) induces an increase in circulating T_reg_ cells (as defined by the FOXP3 and IL-10 mRNA expression of CD4^+^ T cells) in patients with asthma bronchiale [39]. However, such a positive correlation between GC treatment and the number of T_reg_ cells in the peripheral blood is still disputed. In the mouse, Chen *et al.* could demonstrate that the synthetic GC dexamethasone increased the proportion of T_reg_ cells both in peripheral blood and secondary lymphoid organs [40], [41]. By contrast, Stock *et al.* showed the opposite in a mouse model of asthma [42] as did *Wüst et al.* in a mouse model of multiple sclerosis [43]. In humans, several small *in vivo* studies pointed towards a positive correlation between administration of GCs and the frequency of T_reg_ cells in patients with different autoimmune diseases [44], [45], [46], [47], [48], [49], [50], [51], [52], [53], [54]. However, two recently performed larger studies, both including more than 50 patients with asthma bronchiale or autoimmune connective tissue diseases, respectively, arrived at exactly the opposite conclusion [55], [56]. The fact that these studies do not present a unified picture of the influence of GCs on T_reg_ cells may be explained by two aspects. Firstly, there is a huge heterogeneity in the molecular characterization of T_reg_ cells. Several studies just defined T_reg_ cells as being CD4^+^CD25^high^. However, it is evident by now that many of these cells are not T_reg_ cells but rather activated T cells [57]. Secondly, all but one study analyzed patients with an autoimmune background and it is likely that different autoimmune diseases come along with different levels of impairment of T_reg_ cell frequency and/or function [47], [48], [51], [53]. The only study on healthy donors so far was performed *ex vivo* using mixed PBMC cultures in the presence of dexamethasone, epinephrine and IL-2 [58]. Nonetheless, it has been already shown in mice that IL-2 topple the balance in favor of T_reg_ cells regardeless of GC treatment [40], [41].

Hence, the goal of this study was to determine the influence of short-term GC therapy, as frequently used in different clinical scenarios, on circulating T_reg_ cells in immunologically uncompromised mice and humans *in vivo*.

## Methods

### Animal experiments

#### Mice

C57Bl/6 mice were bred in the animal facility at the University of Göttingen Medical School, kept in individually ventilated cages under specific pathogen free conditions and used at an age of 8–12 weeks. Mice of both sexes were included in the study. Blood samples were taken by bleedings from the tail, or the mice were sacrificed using CO_2_ to obtain the spleen or blood from the heart. All animal experiments were conducted according to ethical standards of humane animal care and approved by the authorities of Lower Saxonia (Niedersächsisches Landesamt für Verbraucherschutz und Lebensmittelsicherheit, LAVES; approval ID: 33.14.42502-04-107.08).

GC treatment was performed by intraperitoneal (IP) injection of 0.8 mg/kg, 4 mg/kg, 20 mg/kg or 100 mg dexamethasone dihydrogen phosphate (Dexa-ratio-pharm®, Ratiopharm, Ulm, Germany) / kg body weight on three consecutive days [43]. Mice treated IP with 100 mg/kg were treated for additional 11 days with oral glucocorticoids with decreasing dosages of water-soluble dexamethasone (Sigma, Taufkirchen, Germany) added to the drinking water (days 4 – 7: 10 mg/l, days 8 – 11: 5 mg/l, days 12 – 14: 1 mg/l). The supplemented drinking water was changed every second day.

#### Lymphocyte analysis

Lymphocytes were isolated from the spleen of the mice on days 0, 3 and 14 by passing the freshly isolated organs through a 40 µm Nylon mesh, washed in FACS-Buffer (PBS with 0.5% BSA and 0.05% NaN_3_), erythrolyzed and counted. Absolute cell numbers in blood were determined by flow cytometric analysis of a defined blood volume. Analysis of splenocytes and blood lymphocytes by six-color flow-cytometry was performed using a FACS Canto II device (BD Biosciences, Heidelberg, Germany) in combination with FlowJo software. Monoclonal antibodies directed against mouse leukocyte specific molecules were obtained from BD Biosciences unless otherwise indicated (clone name in brackets): CD3 ε (145-2C11), CD4 (RM4-5), CD8α (53–6.7), CD25 (PC61), TCRαβ (H57-597), GR-1 (RB6-8C5), B220 (RA3-6B2), FoxP3 (FJK-16S; eBiosciences, Frankfurt, Germany). The antibodies were directly labeled with FITC, PE, PerCP, PE-Cy7, Cy5, APC or APC-Cy7, respectively.

#### Analysis of the suppressive activity of T_reg_ cells *in vitro*

CD4^+^CD25^+^ regulatory T (T_reg_) cells and conventional CD4^+^CD25^−^ helper T (T_h_) cells serving as indicator cells were purified from spleens of C57BL/6 mice using the *CD4^+^CD25^+^ Regulatory T Cell Isolation Kit* (Miltenyi Biotech, Bergisch Gladbach, Germany). T_h_ cells (5×10^4^ cells / well) and different amounts of T_reg_ cells were cocultured with γ-irradiated antigen-presenting cells (30 Gy, 10^5^ cells / well) for 48 hrs in RPMI 1640 medium supplemented with 10 % FCS and 1% Pen/Strep in 96-well U-bottom plates. Polyclonal stimulation was achieved by adding Con A (2,5 µg/ml) into the cultures. Water-soluble dexamethasone (Sigma, Taufkirchen, Germany) was added to a final concentration of 5 nM where indicated. Unstimulated and stimulated T_h_ cells and T_reg_ cells alone served as controls. Proliferation was assessed by measuring ^3^H-TdR (Hartmann Analytics, Braunschweig, Germany) incorporation (37 kBq / well) during an additional culture period of 16 hours. The labeled DNA was harvested onto Filtermat A glassfibre filters and the radioactivity was quantified using a MicroBeta^2^ ß-scintillation counter (Perkin Elmer, Rodgau, Germany). In addition, supernatants were collected from cultures after 48 hrs and Interleukin-2 levels were measured using a commercially available ELISA kit (BioLegend, San Diego/CA, USA) according to the manufacturers' instructions.

### Human experiments

#### Patients

16 patients (10 males and 6 females) with acute hearing loss were routinely treated in the Department of Ophthalmology and Otolaryngology of the University Hospital of Würzburg using a modified “Stennert scheme” (only GCs without pentoxifyllin as clinically indicated). All individuals were otherwise healthy and fulfilled the following inclusion criteria: age >18 years (mean patient age was 35.8±13 years), no GCs or other immunosuppressive therapy in the last 8 weeks, no suspected endogenous GC excess and no evidence for any malignant or autoimmune diseases or any acute or chronic infections. All patients gave written informed consent and the study was approved by the ethic committee of the University of Würzburg (Ethik-Kommission der Medizinischen Fakultät der Universität Würzburg, Amendment 1 zu Studien-Nr. 83/05). Prednisolone was administered according to the following regimen: day 1–3: 250 mg intra venous (IV), day 4: 150 mg IV, day 5–9: 100 mg per os (P.O.), day 10–11: 75 mg P.O., day 12: 50 mg P.O., day 13: 20 mg P.O. and day 14: 10 mg P.O.

#### Lymphocyte analysis

Before the first injection of prednisolone (day 0) and at the end of the 14 day treatment period 5 ml EDTA and 20 ml heparinized blood were taken from the patients. The EDTA blood was analyzed in the central laboratory of the University Hospital of Würzburg for a complete blood count with white blood cell differential. The heparinized blood was diluted with 20 ml phosphate buffer saline (PBS). Subsequently, the diluted blood was applied to a Ficoll hypaque® gradient and separated via centrifugation at 400 G for 30 minutes [59]. The peripheral blood mononuclear cells (PBMC) were pipetted from the interface and washed twice with PBS. For a detailed characterization of cellular changes induced by GCs, the PBMCs were stained according to manufacturers' protocols using six different sets of monoclonal antibodies directed against various surface molecules (clone numbers in brackets): Set 1: CD3 (UCHT1), CD14 (M5E2), CD19 (HIB19); Set 2: CD8 (SK-1), CD25 (M-A251), CTLA-4 (BNI3); Set 3: CD4 (RPA-T4), CD25 (M-A251), CD127 (hIL-7R-M21); Set 4: CD4 (RPA-T4), CD25 (M-A251), AITR (eBioAITR); Set 5: CD4/CD 25 (RPA-T4/BC96) and staining control Set 6: no antibodies. Subsequently, the cells which had been stained with the surface markers of sets 5 and 6 were fixed and permeabilized according to the manufacturer's instructions followed by intracellular staining with rat-anti-FOXP3 (PCH101) and the appropriate isotype control antibody (eBR2a). The antibodies used in the sets 1 to 4 were obtained from BD Biosciences (Heidelberg, Germany) and those in the sets 5 and 6 from eBiosciences (Frankfurt, Germany). All antibodies were directly labeled with FITC, PE and APC. Three-color flow cytometry measurements were performed using a FACS Calibur and data analysis was done by using CellQuest pro software (both BD Biosciences, Heidelberg, Germany).

### Statistics

Results are depicted as mean ± standard deviation (SD). For statistical analysis a repeated measures Anova followed by Bonferroni comparison test was performed using Prism v.4.b. software for Macintosh (GraphPad Software Inc., La Jolla, CA, USA). A P value <0.05 was considered statistically significant.

## Results

### Systemic high-dose GC treatment of mice reduces T_reg_ cells numbers in peripheral blood and spleen

**Figure 1:**
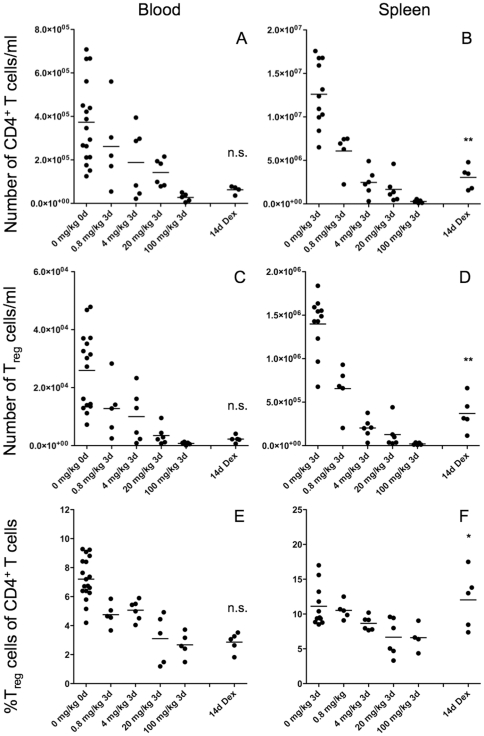
Modulation of CD4^+^ T cells and T_reg_ cells by GCs in mice. Peripheral blood (A, C, E) and spleen (B, D, E) cells from C57BL/6 mice were analyzed by flow cytometry before, 3 days after IP treatment with different dosages of dexamethasone and 14 days after IP treatment with 100 mg/kg dexamethasone followed by oral taper. The absolute numbers of CD4^+^ T cells (A, B) and CD4^+^CD25^high^FOXP3^+^ T_reg_ cells (C, D) were assessed and the relative frequency of T_reg_ cells amongst all CD4^+^ T cells was calculated (E, F); *p<0.05, **p<0.01.

We have chosen a protocol for GC treatment of mice mimicking steroid regimens widely applied to human patients. Whilst therapy of acute relapses in multiple sclerosis patients for example consists of several repeated injection of high-dose GC followed by a taper [60], [61], lower concentrations of GC are used to treat rheumatoid arthritis or asthma patients. Our protocol has been further adapted to consider differences between mice and humans in terms of pharmacokinetics and handling by employing IP injections of dexamethasone [43], [62]. The number of CD4^+^ T cells in the blood of the mice decreased in a dose-dependent manner dropping to levels below 10% of control animals after 3 days of IP treatment with 100 mg/kg dexamethasone (Figure 1A). CD4^+^ T cell numbers remained low even after oral steroid taper (Table 1 and Figure 1A). The number of T_reg_ cells defined as CD4^+^CD25^high^FOXP3^+^ T cells also decreased in a dose-dependent manner after the 3 day of GC therapy and did not recover after an additional 11 days of oral treatment (Figure 1C). Importantly, also the percentage of FOXP3^+^ T_reg_ cells decreased amongst the CD4^+^ T cell population with the GC doses applied, and did not recover at day 14 (Figure 1E).

**Table 1:** Lymphocyte counts and percentages in the blood and spleen of mice before and after GC therapy *in vivo*.

cell numbers (cells / ml ± SD)						
	before therapy	3d after IP therapy with dexamethason (mg/kg)				14d after therapy
Blood		0.8	4	20	100	
CD4^+^ cells	37.3×10^4^±18.8×10^4^	26.2×10^4^±18.9×10^4^	18.8×10^4^±15.7×10^4^	14.2×10^4^±6.2×10^4^	2.8×10^4^±1.8×10^4^	6.3×10^4^±1.8×10^4^
FOXP3^+^ cells*	2.6×10^4^±1.3×10^4^	1.3×10^4^±1.0×10^4^	1.0×10^4^±0.9×10^4^	0.3×10^4^±0.3×10^4^	0.07×10^4^±0.05×10^4^	0.22×10^4^±0.12×10^4^
%FOXP3 in CD4^+^ cells	7.2±1.4	4.8±0.8	5±0.7	3.1±1.7	2.7±0.8	2.9±0.7
spleen		cell numbers (cells / spleen ± SD)				
CD4^+^ cells	12.6×10^6^±3.8×10^6^	6.0×10^6^±2.2×10^6^	2.5×10^6^±1.6×10^6^	1.6×10^6^±1.5×10^6^	0.3×10^6^±0.2×10^6^	3.0×10^6^±1.4×10^6^
FOXP3^+^ cells*	1.4×10^6^±0.3×10^6^	0.7×10^6^±0.3×10^6^	0.2×10^6^±0.1×10^6^	0.1×10^6^±0.1×10^6^	0.02×10^6^±0.01×10^6^	0.4×10^6^±0.2×10^6^
%FOXP3 in CD4^+^ cells	11.1±2.9	10.53±1.3	8.7±1.0	6.7±2.7	6.6±1.9	12.0±.1

* =  as subpopulation of CD4^+^CD25^high^ cells.

‡ =  % out of CD4^+^ cells ± SD.

In the spleen of the same mice the situation was slightly different. Although the absolute number of CD4^+^ T cells also strongly and dose-dependently decreased after the 3 days IP treatment (Table 1 and Figure 1B), the cell numbers partially recovered during the phase of oral GC taper (Figure 1B). The same situation applies to the FOXP3^+^ T_reg_ cells, the number of which decreased after 3 days and slightly recovered after the 14 days of GC treatment (Figure 1D). Consequently, the relative proportion of T_reg_ cells amongst the CD4^+^ T cell population significantly decreased (Figure 1F) after the high-dose systemic GC treatment. Interestingly, the percentage of T_reg_ cells recovered after the additional 11 days of oral taper to values comparable to those before treatment (Figure 1F).

### GC treatment does not impact the suppressive capacity of T_reg_ cells in vitro

**Figure 2:**
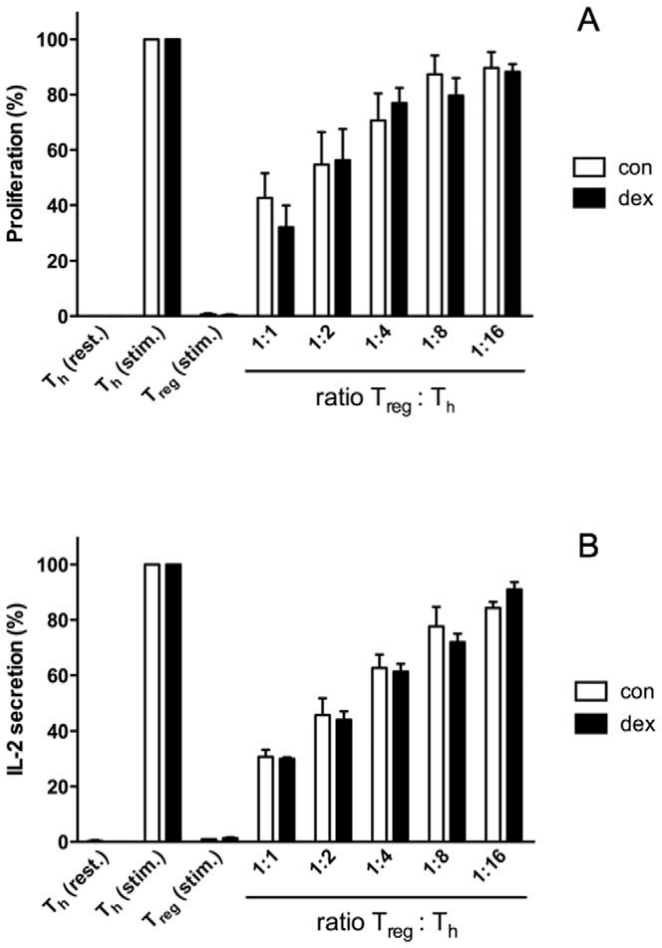
Effect of GC on the function of T_reg_ cells *in vitro*. T_h_ cells (5×10^4^ cells / well) were incubated with T_reg_ cells at different ratios in the presence of irradiated APC (30 Gy, 10^5^ cells / well) and Con A (2,5 µg / ml) for 48 hrs, either with dexamethasone (dex, 5 nM) or without it (con). Resting T_h_ cells as well as T_h_ and T_reg_ cells stimulated with Con A served as controls. Proliferation was determined by ^3^H-TdR incorporation during an additional 16 hrs culture period (A), IL-2 levels were directly measured in the supernatants by ELISA (B). All values were normalized to stimulated T_h_ cells treated with or without Dex, respectively. In both panels the combined data of three independent experiments are depicted.

Besides altering the absolute or relative numbers of T_reg_ cells in blood and peripheral lymphoid organs, it is also conceivable that GC impact the suppressive capacity of T_reg_ cells. This functional characteristic is best studied *in vitro*. Furthermore, a concentration of 5 nM dexamethasone was chosen since pilot experiments had revealed that this already leads to a significant regulation of T cell function while at the same time proliferation is only slightly affected (data not shown). To assess whether GC alter the functional properties of T_reg_ cells *in vitro*, we performed a standard suppression assay in the absence or presence of dexamethasone by adding decreasing amounts of T_reg_ cells to ConA-stimulated T_h_ cells serving as indicator cells. The activity of the T_h_ cells was assessed by analyzing proliferation on the basis of ^3^H-TdR incorporation (Figure 2A) as well as by determining IL-2 production (Figure 2B). Importantly, addition of dexamethasone into the culture did not significantly alter the suppressive capacity of T_reg_ cells, neither in terms of proliferation (42.7±15.5 % at 1∶1 T_reg_:T_h_ ratio in control cells vs 32.0±13.7 % in cells treated with dexamethasone; n.s.) nor in terms of IL-2 secretion (30.7±4.5 % at 1∶1 T_reg_:T_h_ ratio in control cells vs 30.0±1.0 % in cells treated with dexamethasone; n.s.) (Figure 2). This suggests that - at least *in vitro* - GCs do neither positively nor negatively influence the functional properties of T_reg_ cells.

### GCs have little impact on the relative frequency of circulating T_reg_ cells in immunologically uncompromised human subjects

The 14 days of prednisolone administration to human subjects, none of which suffered from immunological diseases, induced a doubling of circulating leukocytes (Table 2). As expected, this was mainly attributed to a significant increase in circulating neutrophils. In addition, the number of monocytes also doubled and the number of lymphocytes increased by 30%, while the numbers of basophils and eosinophils remained unaltered. Of note, the frequency of each cell type within the leukocyte population remained largely unchanged with the exception of the eosinophils that decreased slightly (Table 2).

**Table 2:** Blood lymphocyte counts in immunocompetent human subjects before and after GC therapy *in vivo*.

	cell numbers (cells/ml ± SD)			frequency (% ±SD)		
cell type	before therapy	after therapy	p value	before therapy	after therapy	p value
leukocytes	7.3±3.0×10^6^	13.0±4.2×10^6^	p<0.001	-	-	-
neutrophils	4.6±2.6×10^6^	9.3±3.7×10^6^	p<0.001	60.4±11.6#	68.0±10.3#	n.s.
basophils	1.7±4.0×10^4^	7.0±3.0×10^3^	n.s.	0.2±0.5#	0.1±0.3#	n.s.
eosinophils	1.4±1.4×10^5^	1.3±1.0×10^5^	n.s.	2.5±2.6#	0.9±0.7#	p<0.05
monocytes	5.0±1.5×10^5^	8.5±2.6×10^5^	p<0.001	7.5±2.3#	6.5±1.7#	n.s.
lymphocytes	2.0±0.7×10^6^	3.0±1.3×10^6^	p<0.01	28.7±8.5#	24.0±9.6#	n.s.
CD3^+^ cells	1.5±0.6×10^6^	2.3±1.0×10^6^	p<0.001	58.0±11.0§	58.0±9.0§	n.s.
CD8^+^ cells	4.0±2.5×10^5^	5.8±3.1×10^5^	p<0.001	24.5±9.0$	24.3±7.5$	n.s.
CD4^+^ cells	11.0±4.0×10^5^	18.0±7.7×10^5^	p<0.001	75.5±9.0$	75.7±7.5$	n.s.
FOXP3^+^ cells*	6.2±3.5×10^4^	8.2±4.7×10^4^	p<0.05	4.0±1.5‡	3.4±1.5‡	p<0.05
AITR^+^ cells*	8.9±6.8×10^3^	10.0±9.0×10^3^	n.s.	0.6±0.4‡	0.5±0.3‡	n.s.
CD127^low^ cells*	5.6±2.4×10^4^	10.0±6.0×10^4^	p<0.001	4.0±1.3‡	5.0±3.0‡	p<0.05
CTLA4^+^ cells*	1.6±0.7×10^5^	3.0±2.0×10^5^	p<0.05	13.8±11.5‡	15.6±12.5‡	n.s.

* =  as subpopulation of CD4^+^CD25^high^ cells.

# =  % out of leukocyte cells ± SD.

§ =  % out of PBMC ± SD.

$ =  % out of CD3^+^ cells ± SD.

‡ =  % out of CD4^+^ cells ± SD.

**Figure 3:**
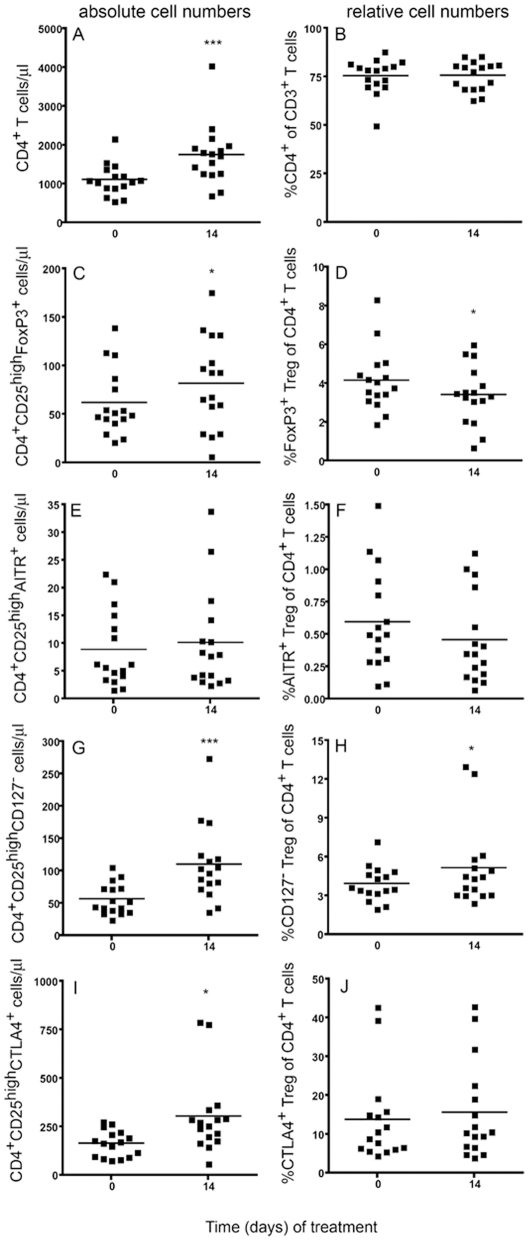
Modulation of CD4^+^ T cells and T_reg_ cells by GCs in humans. Peripheral blood cells from acute hearing loss patients before and 14 days after prednisolone treatment were analyzed by flow cytometry and the absolute numbers (A, C, E, G, I) and the frequency (B, D, F, H, J) of CD4^+^ T cells (A, B) and T_reg_ cells (CD4^+^CD25^high^ and FOXP3^+^ (C, D), AITR^+^ (E, F), CD127^low^ (G, H) or CTLA4^+^ (I, J)) were assessed; *p<0.05, ***p<0.001.

**Figure 4:**
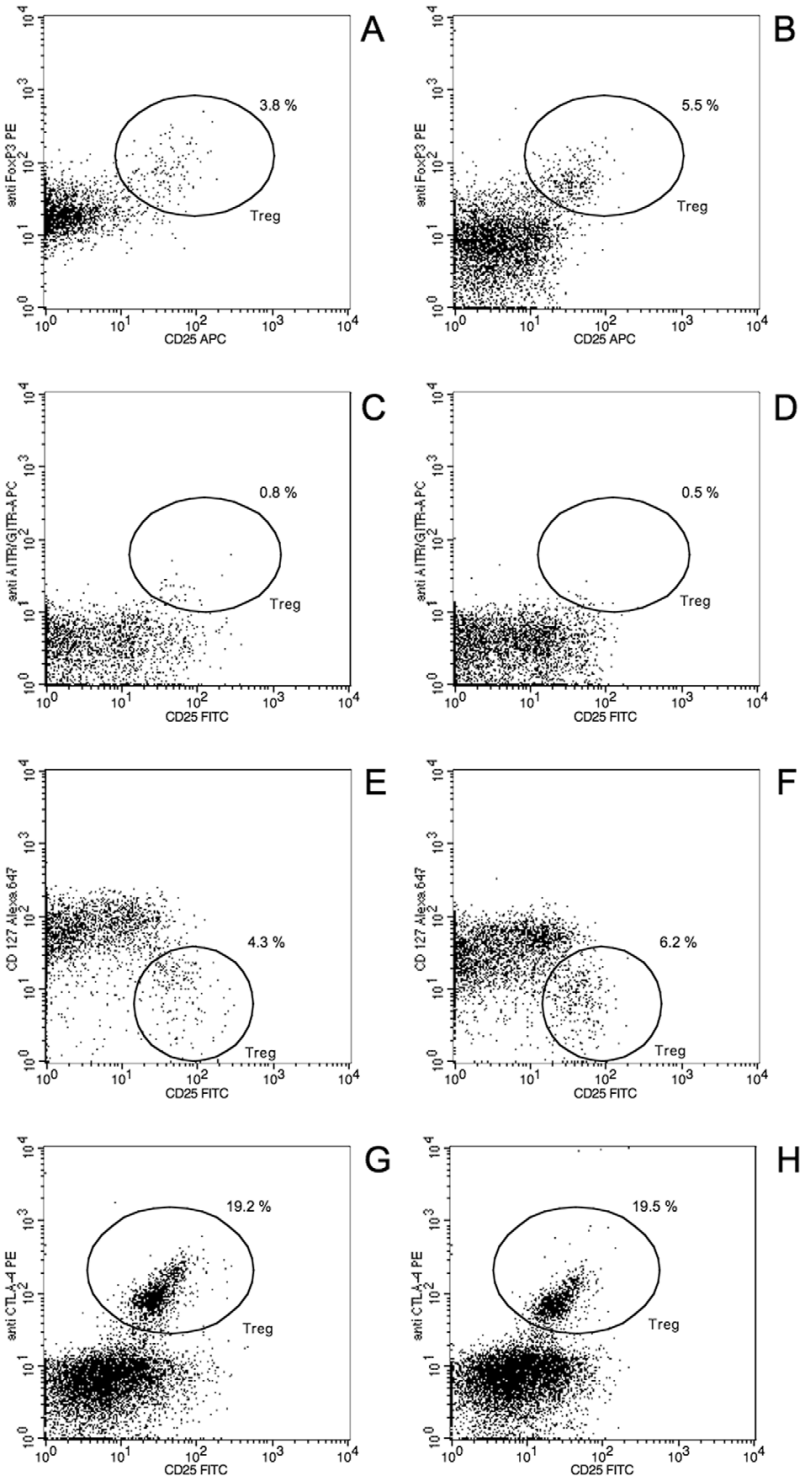
Flow cytometric analysis of T_reg_ cells according to different markers. Peripheral blood from one representative hearing-loss patient treated for 3 days (A, C, E, G) and 14 days (B, D, F, H) with glucocorticoid regimen was analyzed for the presence of regulatory T cells according to the following markers: CD4^+^ CD25^high^ and FOXP3^+^ (A, B), AITR^+^ (C, D) CD127_low_ (E, F) and CTLA4^+^ (G, H). Only CD4^+^ cells are depicted and the percentages indicate the relative frequency of T_reg_ cells within this subpopulation.

With regard to the T cell population, the 14 days of GC administration induced a 1.5-fold increase in the number of CD3^+^ T cells from 15±5.8×10^5^ to 23±10×10^5^ cells/ml (p<0.001; Table 2) due to an increase in CD8^+^ T cell numbers (4±2.5×10^5^ to 5.8±3.1×10^5^ cells/ml; p<0.001) as well as CD4^+^ cell numbers (11±4.0×10^5^ to 18±7.7×10^5^ cells/ml; p<0.001; Figure 3A). In contrast, the relative frequency of all T cell subpopulations remained unaffected (58±11 to 58±9.0% T-cells in PBMC; 24.5±9.0% to 24.3±7.5% CD8^+^ T cells and 75.5±9.0% to 75.7±7.5% CD4^+^ T cells within the T cell population; Figure 3B). The T_reg_ cells, being a subpopulation of the CD4^+^ T cells, followed the positive trend in absolute cell numbers, however, depending on their molecular characterization, the amplitude differed (Figure 4). The number of T_reg_ cells increased only slightly when defined as FOXP3^+^ (62±35×10^3^ to 82±47×10^3^ cells/ml; p<0.05; Figure 3C and 4A, B) or AITR^+^ (8.9±6.8×10^3^ to 10±9.0×10^3^ cells/ml; n.s.; Figure 3E and 4C, D), whereas the CD127^low^ and the CTLA4 T_reg_ cells doubled from 56±24×10^3^ to 100±60×10^3^ cells/ml (p<0.001; Figure 3G and 4E, F) and from 160±70×10^3^ to 300±200×10^3^ cells/ml (p<0.05; Figure 3I and 4G, H), respectively. The frequency of the T_reg_ cells amongst the CD4^+^ T cells also varied depending on the molecular characterization. Whereas the frequency of FOXP3^+^ and AITR^+^ T_reg_ cells within the CD4^+^ T cell population slightly decreased after GC treatment from 4.0±1.5 to 3.4±1.5% (p<0.05; Figure 3D) and 0.6±0.4% to 0.45±0.3% (n.s.; Figure 3F), respectively, their percentages did not increase in a relevant manner from 4.0±1.3% to 5±3.0% (p<0.05; Figure 3H) and from 13.8±11.5% to 15.6±12.5% (n.s.; Figure 3J) when the T_reg_ cells were defined as CD127^low^ and CTLA4^+^, respectively.

## Discussion

T_reg_ cells exert potent suppressive effects on the immune system through a plethora of different mechanisms. It has been previously reported that the frequency of T_reg_ cells is essential for the prevention of exaggerated inflammation and autoimmunity [24], [25], and some studies suggested that T_reg_ cells are induced by GCs both in mice and humans [41], [50]. It was hypothesized that this is due to the differential responsiveness of this specialized T cell subpopulation to GCs [40]. However, the respective experiments were mostly performed not as a monotherapy but rather in combination with potent T-cell inducers like IL-2 or vitamin D3, or they were performed on vaccinated or immunodefficient mice [41], [63]. To reassess the impact of GC therapy on T_reg_ cells in mice in the context of steroid monotherapy, we applied a protocol with increasing dosages of 0.8 to 100 mg/kg dexamethasone IP for 3 days. This therapy led to a dose dependant decrease of CD4^+^ T cells in peripheral blood and spleen. Surprisingly, the decrease in the number of T_reg_ cells was even more pronounced than the number of total CD4^+^ T cells. This is in agreement with the data of *Stock et al.* [42] who show in a mouse asthma model that GCs induce T_reg_ cell deficiency, which aggravates the long-term course of the allergic disease. It is also in line with the data of *Wüst et al.* [43] who reported a relative decrease of T_reg_ cell numbers in a mouse model of multiple sclerosis following high-dose GC therapy. During the additional 11 days of oral taper (from 10 mg/l to 1 mg/l) the low T cell numbers and frequencies in the blood persisted whereas the number of CD4^+^ T cells in the spleen slightly recovered and the proportion of T_reg_ cells even reached similar levels compared to before the therapy. The observed differences between peripheral blood and spleen might suggest that while under GC therapy no new T_reg_ cells are being produced by the thymus, they are constantly generated *de novo* in the secondary lymphatic organs. This corroborates data showing that GCs induce an immature dendritic cell (DC) phenotype in the periphery capable of transforming naïve T cells into T_reg_ cells [64]. We therefore postulate that high-dose CG therapy in the mouse induces massive apoptosis of CD4^+^ T cells, that T_reg_ cells show an increased sensitivity to GCs, and that a prolonged lower dose of GCs favors their tolerogenic DC-induced regeneration in the periphery.

Our finding that the absolute and relative numbers of T_reg_ cells in blood and spleen are decreased by GC treatment *in vivo* does not necessarily exclude that the functional properties of the T_reg_ cells are altered. However, we found that the suppressive capacity of T_reg_ cells was indistinguishable in the presence or absence of GC at least *in vitro*. Neither inhibition of T_h_ cell proliferation nor repression of their IL-2 production by T_reg_ cells was different when dexamethasone was added to the cultures. Although we cannot fully exclude that T_reg_ cell function *in vivo* is affected by GC treatment, our *in vitro* results nevertheless strongly argue that GC do not impact the functional properties of T_reg_ cells directly.

In the human subjects, 14 days of prednisolone administration in a dosage used for the treatment of many common diseases led to strong blood leukocytosis, mainly due to the already described increase in circulating neutrophils and monocytes but to a minor extent also in lymphocytes. Of note, T cells as well as their CD4^+^ and CD8^+^ subpopulations increased at the same rate. On average, the numbers of CD4^+^CD25^high^ T_reg_ cells increased independently of additional markers used for their identification. However, this change was only significant when using FOXP3^+^, CD127^low^ and CTLA4^+^ as definition of T_reg_ cells but not AITR^+^. Similarly, the frequency of FOXP3^+^ T_reg_ cells within the CD4^+^ T cell population decreased slightly, whereas the relative number of CD127^low^ T_reg_ cells increased and the one of AITR^+^ and CTLA4^+^ T_reg_ cells showed no relevant changes concerning the T_reg_/CD4^+^ T cell ratio. We therefore conclude that GCs have no relevant impact on the frequency of circulating T_reg_ cells. However, our study also indicates that the outcome of the analysis of T_reg_ cells in peripheral blood of human subjects depends - at least in part - on the markers used for their molecular characterization. This could explain - amongst others - the partially contradictory results published before. Considering FOXP3 expression, the most accepted marker of T_reg_ cells, in our human cohort, the GC taper treatment seems to induce an increase in T_reg_ cell numbers but at the same time an even stronger increase in CD4^+^ T cells. This even results in a significant reduction of the relative frequency of T_reg_ cells as compared to the rest of the circulating CD4^+^ T cells and thereby confirms our results obtained in mice. Thus, our findings call the hypothesis that GCs exert some of their immunosuppressive capacity by inducing T_reg_ cells into question. Therefore, it is tempting to speculate that GCs rather modify cell-cell interactions of T_reg_ cells with other immune cells than increasing their frequency.

Our results are seemingly in contrast to several previously published *in vivo* studies that showed an increased number of T_reg_ cells and, even more importantly, an elevated T^reg^/CD4^+^ T cell ratio in humans treated with GCs [44], [45], [50]. However, as mentioned before, most of these studies were performed in patients either suffering from hyperimmune or autoimmune diseases [51], [52], [53], [54]. Interestingly, the same studies show a deficiency of T_reg_ cells in the same patients, probably due to thymic dysfunction, and thus the GC treatment seems to only restore their numbers to levels of healthy subjects. Based on our data we therefore hypothesize that GCs induce a tolerogenic environment in these patients leading to the generation of T_reg_ cells in the secondary lymphatic organs which compensates for the thymic dyfunction by creating an apparent advantage of the T_reg_ cells as compared to the rest of the T cells. However, the reconstitution of the T_reg_ cells will last only until the therapy ends and the *de novo* generated T_reg_ cells are recycled, which might explain the short-lived benefit and the high relapse rate described after GC therapy [65].

Our study has strengths and limitations. Firstly, the individuals of our study cohort consisting of patients with acute hear loss are most likely immunocompetent although the exact cause of the acute hear loss is unknown [66], [67]. Secondly, the dosage of GCs in our prospective study was chosen rather arbitrarily and might have impact on the results. Of note, 5 of 7 patients tested on day 14 showed impaired adrenocortical function as measured by ACTH stimulation test (data not shown). In the case of our mouse study, we administered a regimen consisting of increasing doses of dexamethasone [43], whereas in the human study we used the standard dosage for hear loss, which is also utilized for several autoimmune diseases such as lupus erythematosus [68] or idiopathic thrombocytopenic purpura [69]. Thirdly, we analyzed only selected time points and it thus remains open whether the effects that we see are only transient and would be different after shorter or longer time periods of GC treatment. Finally, we used several markers for T_reg_ cell characterization, acknowledging the fact that there is still no consensus on the best definition of T_reg_ cells in humans. However, despite the results being slightly different between each T_reg_ cell characterization, the effect on the relative frequency of circulating T_reg_ cells in general is weak.

In conclusion, short-term GC therapy did not induce the expected increase in the frequency of circulating T_reg_ cells, neither in immunocompetent human subjects nor in mice. In the same time GC treatment *in vitro* did not have any direct effect on the functional ability of the T_reg_ cells. Thus, it is doubtful whether GCs exerts their immunosuppressive effects via influencing the functionality and the (relative) number of T_reg_ cells in blood and spleen.
